# Anthelmintic Resistance to Pour-On Eprinomectin Against Gastrointestinal Strongyles and Effects on Production Parameters in Early-Lactating Dairy Goats

**DOI:** 10.3390/vetsci12111088

**Published:** 2025-11-15

**Authors:** Luisa Rambozzi, Benedetta Torsiello, Roberta Formisano, Mario Pasquetti, Anna Rita Molinar Min, Mauro Giammarino, Luca Battaglini, Martina Sangrali, Manuela Renna

**Affiliations:** 1 Department of Veterinary Sciences, University of Torino, 10095 Grugliasco, TO, Italymanuela.renna@unito.it (M.R.); 2 Department of Agricultural, Forest and Food Sciences, University of Torino, 10095 Grugliasco, TO, Italy; benedetta.torsiello@unito.it (B.T.);; 3 Department of Prevention, Asl TO3, Veterinary Service, 10045 Piossasco, TO, Italy; 4 Veterinary Practitioner, Agrilab S.r.l., 12044 Centallo, CN, Italy; martina.sangrali@gmail.com

**Keywords:** anthelmintic resistance, eprinomectin, caprine milk, *Haemonchus*, *Teladorsagia*, *Trichostrongylus*

## Abstract

Gastrointestinal strongyle infections are a common challenge in dairy goat farming, potentially impacting animal health and milk production. We, therefore, conducted a study to evaluate the efficacy of pour-on eprinomectin (EPM) in early-lactating dairy goats naturally infected with gastrointestinal strongyles and to verify the related effects on milk yield and quality. A total of 42 pluriparous Camosciata delle Alpi goats, from two farms (F1 and F2), were involved in the trial. In each farm, the goats were divided into two groups: untreated (CONTROL) or treated (EPM, 1 mg/kg BW). Following the treatment, faeces were individually collected weekly for one month, subjected to copromicroscopic analysis, a faecal egg count reduction (FECR) test, and coproculture; according to the same time schedule, individual milk yield was recorded, and individual milk samples were collected and analysed (fat, protein, lactose, and somatic cell count). Data were statistically analysed by a mixed-model procedure for repeated measures over time. The low efficacy of EPM was demonstrated at all experimental times; overall FECR percentages (90% CI lower and upper bounds) were 39.00% (30.12–50.53) for F1 and 38.82% (30.08–50.10) for F2. Coprocultures allowed the identification of larvae of the genera *Haemonchus*, *Teladorsagia*, and *Trichostrongylus*. Goats treated with EPM showed a lower prevalence of *Teladorsagia* and *Trichostrongylus* larvae, and higher prevalence of *Haemonchus* larvae, compared with CONTROL goats (*p* < 0.001). The treatment did not significantly improve milk yield or overall milk composition.

## Simple Summary

Gastrointestinal parasite infections are common in small ruminants and can negatively affect their health and production performance. This study evaluated the efficacy of topical eprinomectin administration against gastrointestinal strongyles in early-lactating dairy goats. A total of 42 goats from two farms were included in this study. In each farm, goats were divided into two groups, one receiving the treatment and an untreated group, used as a control. Following the treatment and for 1 month, we collected individual faeces and analysed them in terms of parasite enumeration and taxonomical identification. We also recorded milk yield and analysed milk samples for their fat, protein and lactose contents, and somatic cell counts. In the faeces, we identified eggs of nematodes of the genera *Haemonchus*, *Teladorsagia*, and *Trichostrongylus*. The results showed low efficacy of the treatment towards the genus *Haemonchus* at any experimental time. Consequently, the treatment did not significantly improve milk yield or overall milk composition. These findings suggest the lack of anthelmintic efficacy of topically administered eprinomectin against anthelmintic-resistant goat nematodes of the genus *Haemonchus*.

## 1. Introduction

Gastrointestinal nematodes (GINs) represent a significant health problem in goats, and are responsible for high economic losses in meat and dairy industries, due to slow growth, weight loss, reduced feed consumption, reduced milk production, diminished fertility, and, in the case of massive infections, high mortality rates [1,2,3]. GIN infections often lead to subclinical disease, but symptoms such as anorexia, diarrhoea, weight loss, weakness, rough hair coat, and, in the case of hematophagous species (e.g., *Haemonchus contortus*), anaemia and oedema may be present [4].

The control of GINs mainly depends on the use of anthelmintics [5]. Due to the limited number of active ingredients registered for lactating goats, since the 1980s, there has been intensive and somewhat low rational off-label use of the few available anthelmintic drugs, primarily benzimidazoles and macrocyclic lactones. This practice has led to widespread documented issues of anthelmintic resistance (AR), particularly with highly pathogenic parasitic species [6,7,8].

AR is a significant concern for the small ruminant industry [6] and has been well documented in the three main GIN species affecting goats, namely *H. contortus*, *Teladorsagia circumcincta*, and *Trichostrongylus colubriformis*, all of which also exhibit resistance to multiple anthelmintics [9,10,11]. In some countries, the proportion of resistant GIN strains has become so high that it impedes effective control of parasitic diseases [12].

Goats are more likely to promote AR than cattle or sheep as goats metabolise drugs faster and the spectrum of drugs licenced for this species is limited [13]. Moreover, their browser-like feeding behaviour reduces contact with the invasive parasitic larval stages (L3) present in the environment and, consequently, the possibility of developing immunity against them [13].

Eprinomectin (EPM) is a third-generation macrocyclic lactone characterised by a broad spectrum of endo-parasiticidal and ecto-parasiticidal activity [14]. Macrocyclic lactones irreversibly open glutamate-gated chloride ion channels causing hyperpolarisation, with consequent paralysis of the invertebrate neuromuscular systems [15]. It has been reported that pour-on EPM provides efficacy against helminths at 21 to 28 days after treatment depending on the parasitic species involved [16] and that sustained release formulations provide protection against nematode infections for up to 150 and 120 days in cattle [17] and alpacas [18], respectively. EPM exhibits a favourable milk-to-plasma partition ratio (M/P = 0.17), resulting in low residue in milk [19]. It has been registered in the EU in goats as the only active ingredient with a milk withdrawal time of 0 days, resulting in increasing use. The pharmacological efficacy for goats is highly variable in relation to breed, sex, and physiological stage (lactating/non lactating, age) [20,21], with the risk of underdosing and consequent drug resistance [13,20,22].

Hamel et al. [23] reported that lactation did not interfere with the anthelmintic activity of pour-on EPM. However, these authors worked with goats in the second half of the lactation, while no studies are currently available considering goats in early lactation, a physiological stage when animals usually experience a negative energy balance. Since EPM is a highly lipophilic compound [24], early lactation could be a factor of variability for EPM efficacy [20].

Several reports have also been published concerning the effects of EPM on milk yield and quality in GIN-infected dairy cattle, showing improvements in milk production performance following effective anthelmintic treatments [25,26]. Regarding small ruminants, some data have been published on dairy ewes [27,28], but very few data are available on dairy goats [23,29]. Understanding the efficacy of EPM against GIN infection and the related implications on milk quantitative and qualitative production performance in dairy goat farming is of utmost importance in European countries, where goat milk is commonly used for cheese-making purposes, and the income of goat farmers mainly depends on this activity [30,31].

Therefore, the purposes of this study were to evaluate the efficacy of pour-on EPM in early-lactating goats naturally infected by gastrointestinal strongyles and to highlight any potential beneficial effect of the treatment on goat milk yield and quality.

## 2. Materials and Methods

The study was conducted between April and June 2023. The following criteria were used for selecting farms: (i) breeding Camosciata delle Alpi goats, which is one of the main goat breeds used for milk production purposes in Italy [32]; (ii) adoption of a semi-extensive farming system on owned permanent pastures; (iii) presence of at least 50 pluriparous lactating goats; (iv) on-farm replacement; (v) absence of any endectocide treatment in the periparturient period; (vi) presence of fenbendazole-resistance problems reported by the farm veterinarian; and (vii) no use of EPM in the previous 2 years. Based on the above-mentioned inclusion criteria, two dairy goat farms (F1 and F2) were included in this study. The farms were located in North-Western Italy (44°30′7.114″ N 7°14′38.632″ E; 44°20′11.04″ N 7°46′41.16″ E), and were representative of the typical semi-extensive dairy goat farms present in the Italian Alps, as previously described [31].

At 3 weeks after the start of the grazing season, all the pluriparous lactating goats of the two selected farms were individually subjected to qualitative–quantitative copromicroscopic analysis, their milk yield was recorded, and individual composite samples of morning and afternoon milkings (proportional to milk production recorded per milking) were collected and analysed for gross composition. Based on the obtained results, a total of 42 goats (20 in F1 and 22 in F2) were selected and enrolled in the trial. The selected goats were 36 ± 13.2 and 43 ± 12.8 (mean ± SD) months old and were in early lactation (54 ± 14.8 and 48 ± 15.7 days in milk, in F1 and F2, respectively). In each farm, the goats were divided into two balanced groups according to their gastrointestinal strongyles’ infection [total number of eggs per gram of faeces (EPG); F1: 2895.95 ± 2060.404; F2: 3019.15 ± 2798.615], milk yield (F1: 2.69 ± 0.743 kg/goat × day; F2: 2.09 ± 0.712 kg/goat × day), and milk quality (F1: fat, 34.3 ± 4.73 g/kg; protein, 28.7 ± 4.12 g/kg; lactose, 44.3 ± 1.91 g/kg; somatic cell count (SCC), 1014 ± 1130.8 × 10^3^/mL; F2: fat, 37.9 ± 8.82 g/kg; protein, 28.4 ± 2.52 g/kg; lactose, 44.0 ± 1.57 g/kg; SCC, 877 ± 816.4 × 10^3^/mL). The groups were then randomly assigned to an untreated (CONTROL) group or a treated (EPM) group. At 1 month after the start of the grazing season (T0), the EPM group received pour-on EPM (Eprinex^®^ Multi Pour-On 5 mg/mL—Boehringer Ingelheim Animal Health, **Ingelheim am Rhein**, Germany) at the dosage of 1 mg/kg BW, while the CONTROL groups did not receive any antiparasitic treatment.

Throughout the trial, the goats were allowed to graze during the day, while they were kept indoors during the night. The feeding regime was similar in the two farms and included grazing on natural pastures for 6–8 h per day, supplementary feeding with concentrates and alfalfa hay (up to 1.0 kg for each goat, divided into two meals), and ad libitum access to wheat straw. The goats had unlimited access to water supplies during both the night, when they were kept indoors, and at pasture (through watering tanks). During milking, goats were monitored daily for clinical signs and dag score (0–5 points scale); anaemia was scored weekly (by checking the lower eyelid) using the FAMACHA^©^ card.

### 2.1. Copromicroscopic Analysis

Faecal samples for copromicroscopic analysis (egg counts, coprocultures, and infective larvae harvests/identifications) were collected individually from each goat prior to morning milking, directly from the rectal ampulla, on the day of treatment administration (T0), and then every 7 days for the subsequent 4 weeks (T1, T2, T3, T4).

Faecal samples were collected in disposable sterile gloves, immediately stored at 4 °C in a portable refrigerator, and transported to the Parasitology Laboratory of the Department of Veterinary Sciences of the University of Turin and therein processed within 24 h. Each faecal sample was subjected to quantitative copromicroscopic analysis according to the modified McMaster method using a value of 50 EPG as sensitivity [33].

Positive samples were individually cultured. A minimum of 3 g of each sample was mixed and incubated for 7 days at 27 °C; during this time, the sample was checked periodically and moistened if necessary. Third-stage larvae (L3) were harvested by the Baermann technique for morphometric identification of the parasitic genera, and identified per sample by number, shape, and arrangement of intestinal cells [34].

Treatment effectiveness (%) was evaluated using the faecal egg count reduction (FECR) calculation method, according to the formula proposed by Kaplan et al. [35].

The FECR calculation was performed as follows:

FECR (%) = 100 × (1 − T2/T1)

where T1 is the arithmetic mean of the FEC before treatment and T2 is the arithmetic mean of the FEC after treatment.

Results were interpreted in accordance with Kaplan et al. [35].

### 2.2. Milk Sampling and Analysis

During the sampling occasions, the goats were milked manually. Individual milk yield was recorded using graduated measuring cylinders and individual composite samples of morning and afternoon milkings (proportional to milk production recorded per milking) were collected as previously described [36], according to the same time schedule as described for faecal sample collection. Each milk sample was immediately stored at 4 °C in a portable refrigerator and transported to a laboratory (Agrilab S.r.l., Centallo, CN, Italy) for subsequent analysis of fat, protein, lactose (MilkoScan FT1, Foss Electric, Hillerød, Denmark), and SCC (Fossomatic 360, Foss Electric, Hillerød, Denmark). All analyses were performed in duplicate.

### 2.3. Statistical Analysis

Data were statistically analysed using R software (version R × 64 4.2.2).

The goat was considered the experimental unit. The assumption of normality of the residuals was checked by means of the Shapiro–Wilk test, while the Levene test was used to check for homoscedasticity.

Both parasitological (faecal egg count and number of L3 larvae of each GIN genus identified after coproculture) and milk variables (milk yield, fat, protein and lactose contents and yields, and SCC) were subjected to a two-way repeated measure analysis of covariance (ANCOVA). Within the mixed model, the treatment (TR) was considered as a fixed factor, the time (T) as a repeated measure, the goat as a random factor, and the day of the drug treatment (T0) as a covariate. Furthermore, the interaction between treatment and time (TR × T) was also analysed. When a significant TR × T interaction was detected, a one-way ANOVA was subsequently performed to investigate the differences between the means of all treatment-by-time combinations.

When one or both assumptions were violated, an Aligned Rank Transform (ART) ANOVA for non-parametric analysis was applied.

The results of the statistical analysis are reported as estimated least-squares means. Statistical significance was declared at *p* ≤ 0.05.

## 3. Results

No clinical signs were recorded. Dag and FAMACHA© scores were always <3.

### 3.1. Faecal Analysis

A total of 210 faecal samples were analysed using quantitative copromicroscopic methods.

At any experimental time, on both farms, the percentages of FECR (90% CI lower and upper bounds) were consistently lower than the target efficacy reported by Kaplan et al. [35], reaching a maximum of 39.00% (30.12–50.53%) and 38.82% (30.08–50.10%) in F1 and F2, respectively.

At the individual level, of the 21 animals treated, EPM showed efficacy in only one case (FECR: 100%, in F1); in 7 goats (33.3%, 4 in F1 and 3 in F2), the percentage efficacy was higher than 70%, while in 13 goats (61.9%, 5 in F1 and 8 un F2), it was even lower than 70%. The analyses of the effects of treatment, time, and their interaction indicate a significant decrease in EPG due to EPM treatment in F1 (*p* < 0.001), while the drug did not significantly reduce EPG in F2 (*p* = 0.637). The TR × T interaction was not statistically significant (Table 1).

In the positive samples, a total of 208 coprocultures were prepared, which enabled the identification of L3 larvae from the genera *Haemonchus*, *Teladorsagia*, and *Trichostrongylus*. The percentage distribution of L3 larvae belonging to these three genera, obtained after faecal cultures in the CONTROL and EPM groups, is shown in Table 2. *Haemonchus* spp. comprised the majority of identified L3 larvae (>90% in both farms), while *Teladorsagia* spp. and *Trichostrongylus* spp. were below 6% of the total identified larvae. The percentage distribution of L3 larvae belonging to the genus *Haemonchus* was higher in the EPM-treated goats compared with the CONTROL ones in both F1 and F2 (*p* < 0.001). Regarding the L3 larvae belonging to the genera *Teladorsagia* and *Trichostrongylus*, the EPM-treated goats showed a lower percentage distribution compared with the CONTROL goats in F2 (*p* < 0.001), while a significant interaction TR × T was detected in F1 (*p* < 0.001 and *p* = 0.048, respectively; Table 3).

### 3.2. Milk Yield and Composition

The EPM treatment did not exert significant effects on milk yield or the main milk constituents (fat, protein, and lactose contents and yields) (Table 3). Regarding milk SCC, the EPM-treated goats showed higher values compared with the CONTROL ones in F1 (*p* = 0.002), while no difference between groups was observed in F2. The TR × T interaction was not significant for the considered milk-related parameters (Table 4).

## 4. Discussion

Pour-on EPM was first marketed in the late 1990s at a dose of 0.5 mg/kg BW as a topical endectocide for cattle, with a zero-withdrawal period. In the subsequent years it was largely used off-label (at the same dose) in dairy goats, due to the high level of resistance of GIN to benzimidazoles [37,38]. EPM, which can be administered to goats through topical application at the higher dose of 1 mg/kg BW, with a zero-day milk withdrawal period, has become the main therapeutic option for dairy goats in Europe.

An increasing resistance to EPM has been reported in GIN parasitising cattle [39,40] and sheep [40,41,42]. Some published data is also available for the caprine species [43,44], but limited information can be found for lactating goats and the related effects on milk yield and quality [29]. In the current study, we investigated the anthelmintic efficacy of pour-on EPM administered to dairy goats, also evaluating the potential effects of the drug on milk yield and quality of early-lactating pluriparous subjects in two farms where the farm veterinarian had previously reported AR problems.

Regarding the anthelmintic efficacy, the FECR was lower than the target efficacy reported by the WAAVP [35] at each sampling time, indicating AR to EPM in both farms. With regard to the T3 and T4 sampling times, the EPG values could be overestimated being influenced by possible reinfection, as the goats had access to pasture areas after treatment and throughout the trial. However, it is important to underline that, even if in one farm (F1) the analysis of the FECR indicates a significant decrease in EPG due to EPM, this reduction was not a sign of effectiveness, since also at an individual level EPM, only one goat showed a percentage of effectiveness greater than 90%.

Results of faecal values showed a classical over-dispersion distribution of the GIN faecal egg excretions. Published results reported faecal egg counts of ruminants on pasture, ranging from nearly zero to up to several thousands in some individuals [45].

Despite the high parasitic load, no clinical signs were observed and milk parameters were always in line with those reported in the published literature for this breed in the same geographical area [36,46]. Parasites do not necessarily provoke pathogenic effects in their hosts, their degree of pathogenicity depending on host-related environmental conditions [47]. High egg counts do not necessarily lead to significant diarrhoea [48]. In a study on more than 1500 goats, EPG values from 0 to 19,200 were reported, and in 25.2% of the goats, the EPG value was from 1000 to 5000 [47].

Sajovitz et al. [49], in a recent study on 1195 individual sheep faecal samples in 16 farms, reported EPG values from 0 to 20,175 and concluded that the excretion of strongylid eggs was slightly negatively correlated with the body condition score and slightly positively correlated with the FAMACHA© score, also considering only *Haemoncus*; furthermore, there was no significant correlation between the FEC and dag score.

The number of studies reporting the reduced efficacy of the pour-on EPM formulation compared with oral or injectable formulations of other macrocyclic lactones has notably increased during the last decade [50]. Bordes et al. [10] recorded considerable differences in FECR between moxidectin (100%), injectable EPM (21.5%), and pour-on EPM (−16.7%).

It is surprising that despite the two farms not using EPM for a long time, resistance has emerged. This could be due to the fact that both farms in our study use only farm-owned permanent pastures, resulting in high levels of environmental contamination, also due to the relatively small size of the pasture areas. Unfortunately, resistant parasite populations do not seem to return to susceptibility in the absence of drug exposure, meaning that resistance is essentially everlasting [51], and the anthelmintic resistance is the heritable ability of a parasite to survive anthelmintic treatment; from this definition, it is clearly linked to resistance genes [5].

Furthermore, we have no history of use of any other macrocyclic lactones, which could have induced cross-resistance [52,53]. Considerable resistance to EPM was previously detected even for farms not reporting any previous use of EPM [54]. In a recent study in Slovakia, Babiak et al. [55] reported that pour-on EPM lacked efficacy on 87.5% (7/8) of the tested farms, despite off-label use of EPM in Slovakia being minimal.

The coprocultures showed, in both farms, a complete ineffectiveness of the EPM treatment towards the genus *Haemonchus*, in agreement with available published data [10,44,56]. Otherwise, in both farms, the administration of pour-on EPM to lactating goats appeared to be effective against the genera *Teladorsagia* and *Trichostrongylus,* as also observed in other studies [57,58,59]. However, these results may not be reliable because the prevalence of *Teladorsagia* spp. and *Trichostrongylus* spp. was very low in all the goats in our study.

The predominance of *Haemonchus* spp. compared with other identified GIN genera is likely due to the highest reproductive capacity of individuals (biotic potential), which is very high in this genus of nematodes [60,61]. In addition, *Haemonchus* has a high frequency of heterozygosity and, therefore, a high likelihood of genetic variability [62]. For a parasite, this indicates many attempts at adaptation and, consequently, increased survival chances in response to environmental changes such as, for example, an administered drug [60,61].

The stage of lactation is also a crucial factor to consider when examining the effectiveness of anthelmintics, especially in the case of a lipophilic compound. EPM is a highly lipophilic compound [24]; therefore, early lactation could affect its efficacy [20]. In the current study, the goats were in their first and second months of lactation. A negative energy balance characterises the beginning of lactation due to low feed intake combined with a high energy requirement for milk production, which is then supported by lipolysis of body fat reserves [63,64]. This fundamental aspect could affect pharmacokinetics and, therefore, the antiparasitic action of EPM, as a lipophilic drug. For pour-on EPM, the area under the curve, which is considered an important indicator of the efficacy of macrocyclic lactones, had substantial differences between lactating and dry goats [20]. This can explain why the efficacy results of EPM verified in trials conducted on dry female subjects were more promising, as, in these subjects, the amount of body fat remained more constant [21,65]. Hamel et al. [23] reported an anthelmintic efficacy in lactating goats similar to those observed in adult non-lactating dairy goats treated with pour-on EPM. Indeed, in their study, goats were in the second half of the lactation, thus characterised by a positive energy balance, and a consequent different pharmacokinetic behaviour and pattern of metabolism compared with early-lactating subjects.

Regarding the effects on both the quantitative and qualitative aspects of milk production, in the current study, pour-on EPM administration did not exert any improvement, most probably due to the scarce anthelmintic effectiveness found.

Even if several trials have highlighted the favourable impact of EPM on the milk production performance of grazing dairy cattle [26,66,67], the available data for small ruminants is still scarce. Some authors have reported that, consistent with the results obtained in dairy cows, dairy ewes successfully treated with pour-on or injectable EPM, compared with untreated animals, showed a significant increase in milk yield (up to 8%), probably due to the reported anthelmintic efficacy [27,28,68]. Conversely, regarding the EPM effects on ovine milk quality, no differences were recorded between treated and untreated animals for several parameters, including energy-corrected milk yield, fat, protein and lactose contents, total lactation fat, and protein yields, even if the absolute values were found to be constantly numerically higher in injectable EPM-treated ewes compared with untreated ones [28].

On the other hand, considering the caprine species, studies related to the indirect effects of EPM on milk yield and quality are even lower than those conducted with lactating ewes, especially regarding the topical application of this drug. Alberti et al. [1] found that the extent to which the production performance was negatively influenced by GIN infection was not the same between different goat breeds, with autochthonous ones showing higher resilience to GIN and therefore, less negative impact on milk production performance, compared with cosmopolitan breeds.

In our study, the ineffectiveness of the treatment on milk yield and quality could also be attributable to the higher prevalence of L3 larvae belonging to the genus *Haemonchus* compared with *Teladorsagia* and *Trichostrongylus*. Indeed, *Haemonchus contortus* has been reported to have developed resistance towards several anthelmintics, including macrocyclic lactones [61], which eprinomectin belongs to. Vizcaino et al. [29] reported a significant increase in milk production (+4%) in infected lactating dairy goats in the third to fifth month of lactation treated with injectable EPM. However, it must be emphasised that, in the latter study, L3 larvae belonging to the genera *Teladorsagia* and *Trichostrongylus* were found in higher percentages (65% and 20%, respectively) compared with *Haemonchus* spp. (10%), a factor which may explain the greater effectiveness of the drug against the infection and the significant improvement in milk yield observed by these authors. Consistent with our findings, Vizcaino et al. [29] observed no improvement in goat milk composition following EPM administration. As a final consideration, the observed higher SCC values observed in the EPM-treated goats compared with the CONTROL group in F1 are unlikely to be related to drug administration. In fact, SCC can increase in pasture without intra-mammary infection, due to non-infectious factors, mainly of physical origin [69].

The results obtained in our study show that pour-on EPM had no efficacy in contrasting *Haemonchus* spp. infection in early-lactating dairy goats. Consequently, the EPM treatment resulted in no beneficial effect on the quantitative and qualitative aspects of goat milk production performance compared with an untreated control group.

Considering the significance of AR and its economic impact on goat breeding, further research is advisable to investigate the pharmacokinetics of EPM, focusing on potential variations in early-lactating goats intended for treatment.

## Figures and Tables

**Table 1:** Eggs of gastrointestinal strongyles per gram of faeces (EPG, mean ± SD) counted in the untreated group (CONTROL) and in the pour-on eprinomectin-treated group (EPM) of pluriparous early-lactating Camosciata delle Alpi goats in the two farms.

	**F1**				
	**Treatment**		***p*** **-Value**		
	**CONTROL**	**EPM**	**TR**	**T**	**TR × T**
	(*n* = 10)	(*n* = 10)			
**EPG**	5485 ± 2303	3316 ± 2552	<0.001	0.032	0.281
	**F2**				
	**Treatment**		***p*** **-Value**		
	**CONTROL**	**EPM**	**TR**	**T**	**TR × T**
	(*n* = 11)	(*n* = 11)			
**EPG**	2986 ± 1751	2864 ± 2584	0.637	0.013	0.296

F1, farm 1; F2, farm 2; TR, treatment; T, time; TR × T, interaction between treatment and time.

**Table 2:** Percentage distribution of third-stage larvae (L3, mean ± SD) of gastrointestinal strongyles identified in the untreated group (CONTROL) and in the pour-on eprinomectin-treated group (EPM) of pluriparous early-lactating Camosciata delle Alpi goats in the two farms.

	**F1**				
	**Treatment**		***p-*** **Value**		
	**CONTROL**	**EPM**	**TR**	**T**	**TR × T**
	(*n* = 10)	(*n* = 10)			
*Haemonchus* (%)	92.3 ± 2.78	98.4 ± 2.34	<0.001	<0.001	0.194
*Teladorsagia* (%)	5.85 ± 2.0	1.40 ± 2.0	<0.001	<0.001	<0.001
*Trichostrongylus* (%)	1.90 ± 1.51	0.13 ± 0.5	<0.001	0.373	0.048
	**F2**				
	**Treatment**		***p-*** **Value**		
	**CONTROL**	**EPM**	**TR**	**T**	**TR × T**
	(*n* = 11)	(*n* = 11)			
*Haemonchus* (%)	92.5 ± 5.55	97.6 ± 1.7	<0.001	0.001	0.823
*Teladorsagia* (%)	5.69 ± 5.14	1.83 ± 1.3	<0.001	<0.001	0.150
*Trichostrongylus* (%)	1.72 ± 2.33	0.73 ± 0.89	<0.001	0.038	0.333

F1, farm 1; F2, farm 2; TR, treatment; T, time; TR × T, interaction between treatment and time.

**Table 3:** Effect of treatment × time (TR × T) interaction on the percentage distribution of third-stage larvae (L3, mean ± SD) belonging to the genera *Teladorsagia* and *Trichostrongylus* at each sampling time (T1; T2; T3; T4) in F1.

Treatment				*p*-Value
	Time	CONTROL (*n* = 10)	EPM (*n* = 10)	
*Teladorsagia* (%)	T1	4.68 ± 2.05 ^ab^	0.00 ± 0.0 ^d^	<0.001
	T2	5.66 ± 2.44 ^a^	0.14 ± 0.0 ^d^	
	T3	6.66 ± 1.23 ^a^	2.53 ± 1.7 ^c^	
	T4	6.40 ± 1.6 ^a^	2.93 ± 2.54 ^bc^	
*Trichostrongylus* (%)	T1	2.06 ± 1.50 ^a^	0.00 ± 0.0 ^c^	0.048
	T2	1.90 ± 1.48 ^a^	0.00 ± 0.0 ^c^	
	T3	1.70 ± 1.35 ^a^	0.38 ± 0.7 ^bc^	
	T4	1.93 ± 2.04 ^a^	0.48 ± 0.53 ^bc^	

TR × T, interaction between treatment and time. a, b, c, d: for each genus, means with different superscript letters differ significantly (*p* < 0.05).

**Table 4:** Yield, main constituents, and somatic cell count (mean ± SD) of milk from pluriparous early-lactating Camosciata delle Alpi goats of the untreated group (CONTROL) and of the pour-on eprinomectin-treated group (EPM) in the two farms.

	**F1**				
	**Treatment**		***p*** **-Value**		
	**CONTROL**	**EPM**	**TR**	**T**	**TR × T**
	(*n* = 10)	*(n* = 10)			
Milk yield (kg/goat × d)	2.37 ± 0.56	2.25 ± 0.82	0.397	0.966	0.974
	Milk composition				
Fat (g/kg)	32.3 ± 5.83	31.9 ± 5.99	0.770	0.131	0.991
Protein (g/kg)	26.5 ± 2.53	27.2 ± 3.18	0.978	0.001	0.079
Lactose (g/kg)	44.3 ± 1.96	43.4 ± 2.35	0.102	0.593	0.969
Somatic cells (mL × 10^3^)	913 ± 1175	1654 ± 932	0.002	0.555	0.997
	Component yield				
Fat (g/goat × day)	71.4 ± 18.3	73.5 ± 21.8	0.641	0.419	0.903
Protein (g/goat × day)	60.4 ± 13.2	61.8 ± 18.7	0.699	0.866	0.943
Lactose (g/goat × day)	104 ± 23.0	98.0 ± 34.6	0.357	0.927	0.939
	**F2**				
	**Treatment**		***p*** **-Value**		
	**CONTROL**	**EPM**	**TR**	**T**	**TR × T**
	(*n* = 11)	(*n* = 11)			
Milk yield (kg/goat × d)	1.92 ± 0.58	1.84 ± 0.52	0.322	0.107	0.192
	Milk composition				
Fat (g/kg)	35.2 ± 5.11	35.3 ± 7.4	0.921	<0.001	0.758
Protein (g/kg)	28.2 ± 4.54	27.5 ± 2.92	0.900	<0.001	0.555
Lactose (g/kg)	43.4 ± 1.39	43.6 ± 1.83	0.339	<0.001	0.879
Somatic cells (mL × 10^3^)	1313 ± 1465	1369 ± 1460	0.870	0.004	0.729
	Component yield				
Fat (g/goat × day)	68.2 ± 24.4	67.4 ± 25.6	0.803	0.031	0.120
Protein (g/goat × day)	53.4 ± 16.6	50.1 ± 13.1	0.182	0.074	0.766
Lactose (g/head × day)	84.6 ± 26.3	81.3 ± 22.3	0.361	0.037	0.320

F1, farm 1; F2, farm 2; TR, treatment; T, time; TR × T, interaction between treatment and time.

## Data Availability

The raw data supporting the conclusions of this article will be made available by the authors on request.

## Abbreviations

The following abbreviations are used in this manuscript:

ANCOVA	Analysis of covariance
ANOVA	Analysis of variance
AR	Anthelmintic resistance
ART	Aligned rank transform
BW	Body weight
EPM	Eprinomectin
FAO	Food and Agriculture Organization
FECR	Faecal egg count reduction
GIN	Gastrointestinal nematodes
SCC	Somatic cell count
